# Informing Patients About Placebo Effects: Using Evidence, Theory, and Qualitative Methods to Develop a New Website

**DOI:** 10.2196/resprot.5627

**Published:** 2016-06-10

**Authors:** Maddy Greville-Harris, Jennifer Bostock, Amy Din, Cynthia A Graham, George Lewith, Christina Liossi, Tim O’Riordan, Peter White, Lucy Yardley, Felicity L Bishop

**Affiliations:** ^1^ Psychology Department Faculty of Social and Human Sciences University of Southampton Southampton United Kingdom; ^2^ Institute of Psychiatry, Psychology & Neuroscience King's College London London United Kingdom; ^3^ Centre for Innovation & Leadership in Health Sciences Faculty of Health Sciences University of Southampton Southampton United Kingdom; ^4^ Primary Care and Population Sciences Faculty of Medicine University of Southampton Southampton United Kingdom; ^5^ ZeMedia Southampton United Kingdom

**Keywords:** placebo effect, informed consent, qualitative research, health attitudes, consumer health information

## Abstract

**Background:**

According to established ethical principles and guidelines, patients in clinical trials should be fully informed about the interventions they might receive. However, information about placebo-controlled clinical trials typically focuses on the new intervention being tested and provides limited and at times misleading information about placebos.

**Objective:**

We aimed to create an informative, scientifically accurate, and engaging website that could be used to improve understanding of placebo effects among patients who might be considering taking part in a placebo-controlled clinical trial.

**Methods:**

Our approach drew on evidence-, theory-, and person-based intervention development. We used existing evidence and theory about placebo effects to develop content that was scientifically accurate. We used existing evidence and theory of health behavior to ensure our content would be communicated persuasively, to an audience who might currently be ignorant or misinformed about placebo effects. A qualitative ‘think aloud’ study was conducted in which 10 participants viewed prototypes of the website and spoke their thoughts out loud in the presence of a researcher.

**Results:**

The website provides information about 10 key topics and uses text, evidence summaries, quizzes, audio clips of patients’ stories, and a short film to convey key messages. Comments from participants in the think aloud study highlighted occasional misunderstandings and off-putting/confusing features. These were addressed by modifying elements of content, style, and navigation to improve participants’ experiences of using the website.

**Conclusions:**

We have developed an evidence-based website that incorporates theory-based techniques to inform members of the public about placebos and placebo effects. Qualitative research ensured our website was engaging and convincing for our target audience who might not perceive a need to learn about placebo effects. Before using the website in clinical trials, it is necessary to test its effects on key outcomes including patients’ knowledge and capacity for making informed choices about placebos.

## Introduction

Clinical trials must be conducted in accordance with Good Clinical Practice guidelines [1] and ethical principles espoused in the Declaration of Helsinki [2]. In particular, patients should be fully informed about the interventions they might receive and must give written informed consent. In a placebo-controlled randomized clinical trial this means that all participants should be fully informed about both the new intervention being tested and the placebo control. However, a content analysis of participant information leaflets from United Kingdom–based placebo-controlled trials found that written information about placebos is typically incomplete and at times misleading [3]. For example, despite strong evidence of placebo effects and mechanisms in the scientific literature, [4-7] only 1 of 45 leaflets explicitly stated that patients might experience beneficial effects from the placebo [3]. Using placebos in clinical trials appears to be generally acceptable to patients, but crucially this depends not only on the severity of the condition being treated and available alternative controls, but also the adequacy of informed consent [8]. Furthermore, evidence from surveys and qualitative studies shows that clinical trial participants often have false beliefs about, and partial understanding of, placebos and their possible effects [9-11]. Examples of such false beliefs include the belief that placebo effects are fake, or illusory, and that people who respond to placebos are gullible or foolish [11-13]. This might explain why the inclusion of placebo controls can deter people from volunteering to participate in trials [14]. Overall, it seems that a significant proportion of clinical trial participants might have inadequate understandings of the potential clinical effects of placebo interventions [9-13], thus jeopardizing the ethical validity of informed consent [3] and potentially hampering recruitment [14]. More accurate and complete information about placebos could usefully address this ethical shortcoming, could combat patient anxiety about placebo effects [12], and reduce distress at being debriefed in placebo condition participants [15,16].

This paper describes the development of an educational website about placebo effects. We aimed to create an informative, scientifically accurate, and engaging website that could be used to improve understanding of placebo effects among patients who might be considering taking part in a placebo-controlled clinical trial. We chose to develop a website rather than a traditional printed information leaflet because websites (1) are increasingly popular sources of consumer health [17-20], suggesting that this format reflects consumer preferences, (2) easily incorporate interactive features [21], which can enhance engagement and effective education[22], (3) are easily and cheaply disseminated for widespread access [21] (86% of UK households had Internet access and 78% of adults accessed the Internet daily or almost daily in 2015 [23]), and (4) can be readily adapted and/or tailored to different subgroups [21]. Our intention was to develop a resource that could potentially be used across a large number of clinical trials and/or adapted for use in specific trials. However, because the effects and mechanisms of placebos differ across symptoms and diseases [24], we chose one clinical target to focus on for this version of the website. We chose placebo analgesia as pain is relevant to a large number of clinical conditions and placebo analgesia is a well-documented placebo effect [4,25-27]. The target audience for this website was thus adults experiencing pain symptoms (from any clinical condition).

## Methods

### An Evidence-, Theory-, and Person-Based Approach

**Figure 1:**
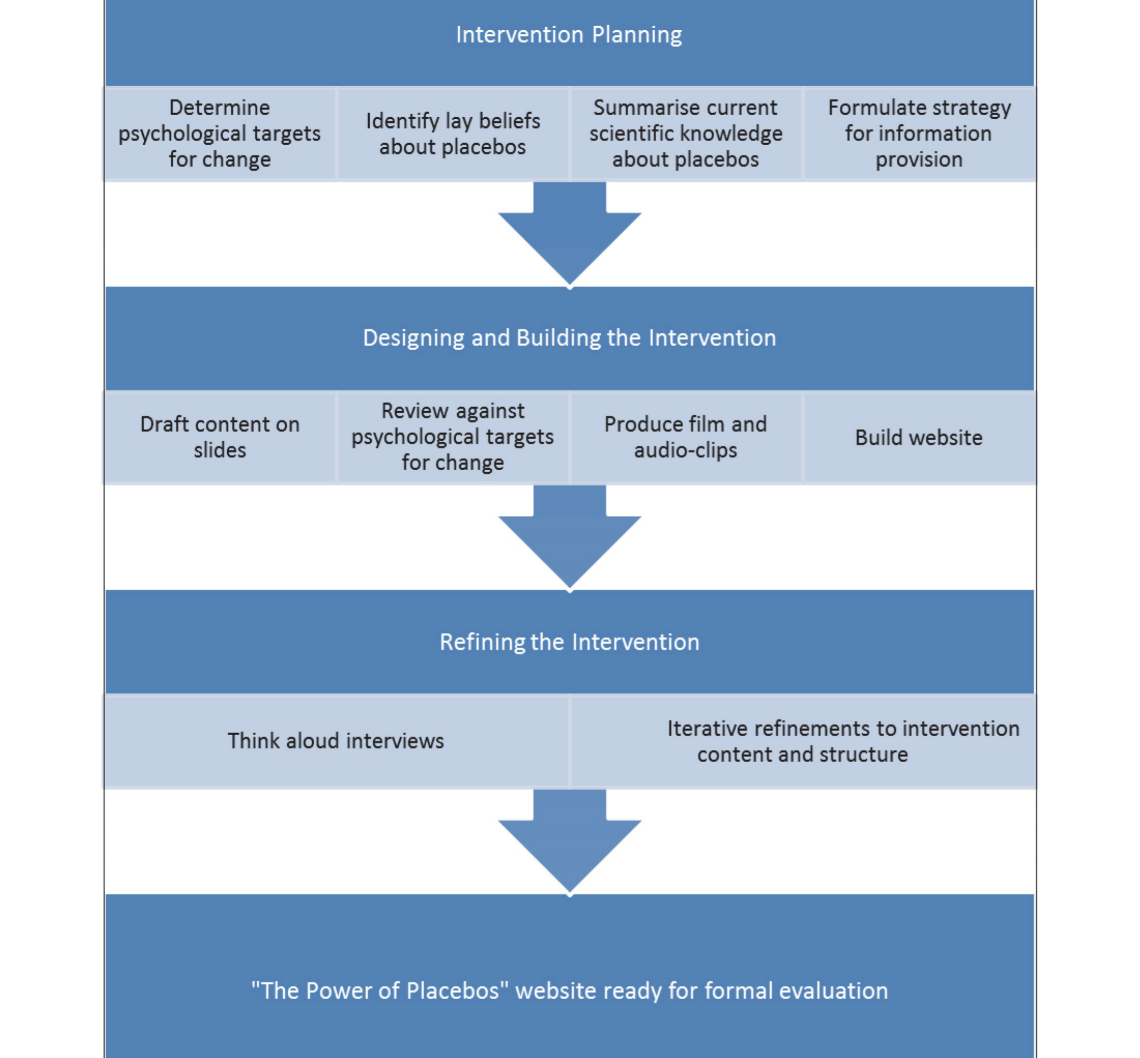
Overview of methods used to develop the website.

We drew on existing evidence and theory, and conducted qualitative research to develop our website using an approach derived from evidence-based, theory-based [28,29], and incorporating elements of person-based [30] intervention development. In the context of a website about placebos, we felt this combined approach was more valuable than any single approach. It was important to use existing evidence and theory about placebo effects to ensure our content was scientifically accurate. Drawing on existing evidence and theory about health behavior was essential to ensure our content would be communicated persuasively to an audience who might currently be ignorant or misinformed about placebo effects. Conducting our own qualitative research was necessary to ensure our website was engaging and convincing for our target audience who might not have a perceived need to learn about placebo effects. Figure 1 provides an overview of the process of developing the website.

### Planning the Intervention

To plan the content and structure of the website we considered four key questions:

What psychological targets are relevant to providing information about placebos? In other words, what would we expect to change as a result of viewing our website?

What do patients typically believe about placebos and placebo effects?

What is the current scientific understanding of placebos and placebo effects?

How should information about placebos and placebo effects be provided to be most effective?

### Psychological Targets

Given current partial understandings, misunderstandings, and poor quality information about placebos [3,9-11], we decided our website should focus on promoting informed choices about placebos. Making an informed choice can be understood as choosing to act in a way that is based on one’s knowledge and one’s values [31-33]. According to this definition, it is incorrect to specify that a particular option is the correct choice for everyone: what counts as the informed choice will differ across equally knowledgeable individuals according to their values. To make an informed choice, a person needs to have an accurate understanding of the options available, have formed an opinion about the options based on their values, and make a decision (or otherwise act in a way) that is consistent with their knowledge and values. We chose to focus on informed choice, rather than informed consent, because the process of informed consent to take part in a placebo-controlled clinical trial would typically involve a face-to-face interaction between trial personnel and potential participants and would involve the provision of trial-specific information (eg, about the trial intervention). Our website is intended to provide a generic resource to educate and inform members of the public who may be considering receiving placebos as part of a clinical trial.

In the context of placebos, an informed choice to receive a placebo (eg, by taking part in a trial) requires knowledge about the possible effects of placebos, a positive attitude to taking placebos, and a decision to take placebos. An informed choice not to receive a placebo requires knowledge about the possible effects of placebos, a negative attitude to taking placebos, and a decision not to take placebos. To promote informed choice our website thus needed to improve people’s knowledge of placebos. Therefore, knowledge about placebos and their effects was our primary target for change.

Making an informed choice to receive placebos can be understood as a volitional behavior, and thus can be modelled using the Theory of Planned Behavior [34]. According to the Theory of Planned Behavior, patients’ intentions to take placebos are driven by attitudes, subjective norms, and perceived behavioral control. These, in turn, are determined by beliefs [34]. Thus, patients will be more likely to make an informed choice to receive placebos if they (1) believe that placebos are effective/good for them and value these effects (attitudes), (2) believe that people whose opinion they value would approve of placebos (subjective norms), and (3) believe that they control whether or not they receive placebos (perceived behavioral control). While we did not want to encourage people to decide to receive placebos (except in the context of an informed choice), we did want to provide information that was consistent with people’s existing cognitive structures. Therefore, we designed the website to address the following: consequences of receiving placebos for the individual (attitudes), other people’s views on placebos (subjective norms), and practicalities around receiving placebos (perceived behavioral control).

### Typical Beliefs About Placebos

To understand typical beliefs about placebos we reviewed qualitative studies of patients’ experiences of placebos in clinical trials. This literature showed that people are interested in but often anxious about or confused by placebos [35,36]. For example, in qualitative studies embedded in acupuncture trials patients described interest or anxiety as to whether they receive placebo or real treatment [37], and the knowledge that they may receive placebo made them doubt any perceived improvements in symptoms [12,13]. Patients conceptualize placebos and their effects in various ways, dominant among which are understandings of placebos: as fake treatments that fool people into thinking they are better; as tools that are necessary for scientific research; and as interventions that have real effects mediated by psychological mechanisms [11]. Patients’ reactions to being told they had been in the placebo arm of one clinical trial included surprise and disbelief. Some worried that they would ‘throw the trial off’ and interpreted their experiences in a way that affirmed their understanding of their illness or emphasized the positive effect of social support from trial staff [15].

Based on these findings, we devised website content to describe the possible (positive and negative) effects and experiences of placebo (‘can it help?’, ‘what is it like?’). The website also addresses common concerns about placebos (‘what concerns me’), debunks myths that placebo responders are malingerers or gullible (‘who does it help?’), and explains the mechanisms underpinning placebo effects (‘how could it work?’).

### Scientific Understanding of Placebo Effects

To ensure scientific accuracy we consulted systematic and narrative reviews and seminal studies concerning the effects and mechanisms of action of placebos [4,6,7,24]. We selected key evidence-based facts about placebo effects to convey in the website including: placebos can relieve pain and stiffness in osteoarthritis [27]; placebos can improve reported pain across a number of painful conditions [25]; and placebos can elicit side effects in what is called a ‘nocebo’ effect [38] (ie, the negative aspect of the placebo effect that occurs when an adverse event, such as experiencing side effects, is triggered by negative expectations [39]). The website also conveyed that it is difficult to know how many people placebos will work for, but they seem to improve pain for between 26% and 51% of people in placebo studies [6].

The mechanisms of action of placebos have been described in neurobiological, psychological, and anthropological terms. Patients also appear to develop understandings of placebo effects at different levels, with some focusing on psychological processes and others emphasizing social processes [11,15]. To accurately represent the scientific literature and appeal to different patients, we therefore chose to convey a number of different theories of placebo effects, specifically neurobiological pathways [40], expectancy [41], conditioning [42], meaning response [43], and the therapeutic relationship [44].

We also drew on scientific literature when developing material on other aspects of placebos outlined in the previous section. For example, in addressing concerns that doctors have to lie to patients to give them placebos, we drew on evidence about open-label placebo prescribing, suggesting that doctors do not have to lie to patients to elicit placebo effects [5].

### Effective Information Provision

To plan how to provide information effectively to educate people about placebos and thus improve their knowledge, we considered a selection of relevant theories. In particular, we drew on theories of motivation, learning, and attitude formation.

We drew on Self-Determination Theory [45] to plan how to design our website so that it would be maximally engaging for people. Self-Determination Theory distinguishes between intrinsic motivation (eg, curiosity) and extrinsic motivation (eg, payment) as drivers for action; our website relies on intrinsic motivation as we do not anticipate eventual users to receive external rewards for using it. Cognitive Evaluation Theory (1 of 6 ‘mini-theories’ within Self-Determination Theory) elaborates on how social contexts can impact on intrinsic motivation, and suggests that intrinsic motivation can be enhanced by satisfying basic human needs of competence and autonomy [45]. Thus, if the website supports people’s perceptions of themselves as competent and autonomous it should enhance intrinsic motivation and be more engaging. To promote perceptions of competence, we designed easy quizzes (using the word ‘surprise’ rather than ‘wrong’ to give feedback on incorrect answers) and used simple and consistent navigation. To promote perceptions of autonomy, we allowed users as much freedom of choice as possible, for example in terms of the order in which to view different pages.

Educational theory suggests that people have different learning styles [46]; therefore, we decided to provide information in different formats: written text, photographs and images, audio clips, and film. We used quizzes to engage readers in active learning [47] and to test readers’ knowledge (tests are an effective means of improving learning [48]). We also considered to whom to attribute different sources of information. According to Social Learning Theory, when we identify with another person (a ‘model’) and perceive them to be competent and similar to us, we may learn from observing them [49-51]. Therefore, we decided to use actors to narrate first-person accounts of patients’ experiences of taking placebos, and to choose actors of various ages, genders, and ethnicities. We drew on qualitative studies of real patients’ experiences in clinical trials to develop the first-person accounts [11-13,15].

While our focus was on educating people by providing accurate information about placebos, attitude formation and change processes might also occur in response to information provision. According to leading theories of attitude change, there are both central and peripheral routes to attitude formation and change [52]. Central routes are well-described by the Elaboration Likelihood Model and entail highly-motivated individuals engaging in an effortful way with substantive messages, assessing new information in relation to previously held beliefs, and coming to a reasoned conclusion [53]. Peripheral routes, as described by the Heuristic Systematic Model of persuasion, entail the use of simple heuristics or ‘rules of thumb’ based on superficial cues such as source credibility and number of arguments presented [54,55]. To encourage the development of more informed attitudes toward placebo effects, we described scientific evidence to support our substantive message that placebos can have effects, presented multiple scientific theories about how placebos produce effects, and bolstered the credibility of the message source by describing the website and study authors’ academic credentials.

We followed guidance for developing patient-focused health information by considering five key issues: information needs, accessibility, quality, readability and comprehensibility, and usefulness [56]. To ensure we addressed different needs for information, we allowed readers a choice about whether to access basic or more complex information. In other words, we used simple text to convey basic information about a topic (based on the literature), then offered a click-through to a more detailed evidence summary describing a specific study or review, and then offered another click-through to access the actual scientific paper. To provide access to scientific evidence we wrote accurate text, evidence summaries, and in some cases, provided full papers. We considered color blindness and dyslexia in our choice of colors and formatting; for example, using clear, plain text and consistent formatting throughout. We also ordered the items on the pages to make them more accessible for people using text-reader software. To ensure we provided high-quality information we used peer-reviewed publications and reviews. To enhance readability we wrote in short sentences with simple sentence construction and used readability indices to guide our writing. To enhance usefulness, we provided information on relevant topics (according to the literature on patients’ views of placebos), included patient representatives in the research team, and conducted a think aloud study to elicit users’ feedback.

### Designing and Building the Intervention

We used the insights gained during the planning phase to write the content and map out the initial structure of our website using PowerPoint slides. Content was based on published evidence, as described above. To ensure content was relevant to our primary targets for change (knowledge, informed choice) and mapped onto existing cognitive structures (attitudes, subjective norms, perceived behavioral control), we reviewed each draft page for relevance to these targets and structures. We then built the website using LifeGuide open source software to facilitate the design and scientific testing of Web-based behavior change interventions [57].

Audio clips were produced for first-person narratives about the phenomenology of placebo effects. A film was scripted and produced to illustrate a placebo effect in an experimental context (using a cold pressor task) and to describe the effects and mechanisms of action of placebos. As a visual medium that lends itself well to linear narrative, film provides an unrivalled, vivid view of the world and may capture events, people, and performances with detail and richness. Koumi [58] suggested a number of considerations for writing instructional film scripts including: the ‘hook’ (an element which captures viewers’ attention), asking questions, synergy between image and narration, clarity of argument, audio/visual cues to denote changes, and argument consolidation. The film we produced integrated these steps through the use of animated infographic, live action, interview, and narration to reinforce key messages. While there is no hard-and-fast rule as to how many messages a video can contain, studies show that people can store approximately four discrete ‘chunks’ of information in short-term memory [59], and some writers advise that a 30-minute video can comfortably elucidate three essential points in some detail. As the placebo video has a running time of 4 minutes 20 seconds, the script was written to focus on two key points: that placebos have significant, measurable, positive effects on health, and that these effects are present in conventional treatments.

Korakidou and Charitos [60] asserted that film involves viewers on an emotional as well perceptual level, employing empathy to facilitate engagement and retention. In addition, in productions that include the spoken word it is believed that viewers prefer and empathize more readily with conversational language that is easily understood [61]. To facilitate this, the video used live action sequences to encourage viewers to imagine themselves in similar situations, and simple, jargon free narration to ease comprehension and reduce cognitive load.

### Think Aloud Study

A small qualitative study during this phase informed the final content and structure of the website. Ethical approval was obtained from the host institution (reference: ergo id 10933) and all participants gave written informed consent. Posters and Web-based advertisements were used to recruit 10 participants from the host institution (9 female, a mix of staff and students, aged 19-35 years, 4 with musculoskeletal pain). They worked through the website in the presence of the researcher, speaking aloud their thoughts and answering specific, probing questions (eg, “what do you think this page is about?”, “What did you think about navigating through the site?”). The mean interview duration was 32 minutes (range, 22-40) and interviews were audio-recorded. Interviewees’ comments were reviewed and coded according to the topic to which they referred using deductively derived codes based on aspects of the website (eg, “placebos in clinical practice”, “patients’ stories”) and inductively derived codes for comments that did not relate directly to specific contents (eg, “technical terms”). Comments were further categorized as primarily related to content, style, or navigation. Two researchers were involved in interpreting interviewees’ comments, which avoided an idiosyncratic focus on particular issues and enabled discussion of which comments to prioritize when deciding on modifications to the website (eg, in cases where interviewees provided divergent and/or conflicting perspectives). Interviews proceeded iteratively, with early interviews being analyzed first and used to inform changes to the website that were then presented in later interviews.

## Results

Comments from participants in the think aloud study highlighted aspects of the website that participants felt were engaging as well as occasional misunderstandings and stylistic and structural features that were off-putting and/or confusing, and thus needed improving. Elements of content, style, and navigation were therefore modified to improve participants’ experiences of using the website. Table 1 presents selected quotes from participants, illustrating how their perspectives were used to inform website modifications. Content changes included: elaborating on ‘mind-body healing processes’ as participants found this vague and wanted more information about how placebo effects might work; adding reassurance that pain is still real even if it reduces with placebo; adding specific details and evidence from scientific papers about how many doctors use placebos; and adding an example to address participants’ concerns about how doctors can justify using placebos clinically: “For example, sometimes doctors may listen to a patient's chest, even when it is not essential for making a medical diagnosis, because it can be reassuring for the patient.” Stylistic changes included: choosing photos (for patient’s stories) that look more realistic as participants felt some looked like stock photos, and thus lacked authenticity; ensuring alignment and consistent logo placement throughout as participants noticed minor misalignments and saw them as unprofessional, reducing the credibility of the website; and modifying patients’ stories to always use convincing lay language, and thus avoid the off-putting impression of advertising. Navigation changes included: adding a menu bar to all pages to make each page accessible from any other page, and page buttons changing color once viewed.

**Table 1:** Illustrative Quotes from Participants Used to Refine the Website

Topic	Quote	Modification
Content: information on placebo effects	So it’s basically asking a question that yes it can help with your pain. And obviously there’s been quite a big study that shows that it can definitely relieve pain and stiffness. There’s obviously useful information. (Participant 2)	None needed
Content: patients’ stories	I think it's good to have a case study. I didn't realize you could do placebo surgeries so that's quite interesting. (…) It's nice to have a picture of the person as well. It's good to have the option to read what he says as well, so if you don't want to read it, you can listen to it. (Participant 3)	None needed
Content: technical terms	Maybe explain what ‘mind-body self-healing processes’ are. Um otherwise I think like I understand what it says, yeah. (Participant 1)	Expand on ‘mind-body self-healing’
Content: placebos in clinical practice	What is going through my mind maybe a little bit is maybe kind of if I was to receive the placebo and not actually know. So maybe something that addresses that, how common it is to be given a placebo in the health care sector. (Participant 8)	Add specific details about how many doctors use placebos
Style: patients’ stories	Are these the pictures of the actual people who are talking or are they stock pictures? (Participant 6)	Change photos
Navigation: menu	Like usual websites have everything on the same page, so it’d have a menu at the side that you always see. So is a bit confusing because I'm going back, but I cannot remember what I've clicked on necessarily. It’s all like a flow, you have to go through it, and back again. I think it might be more useful to have a menu at the edge or something. (Participant 5)	Add a side menu bar that is always available

**Figure 2:**
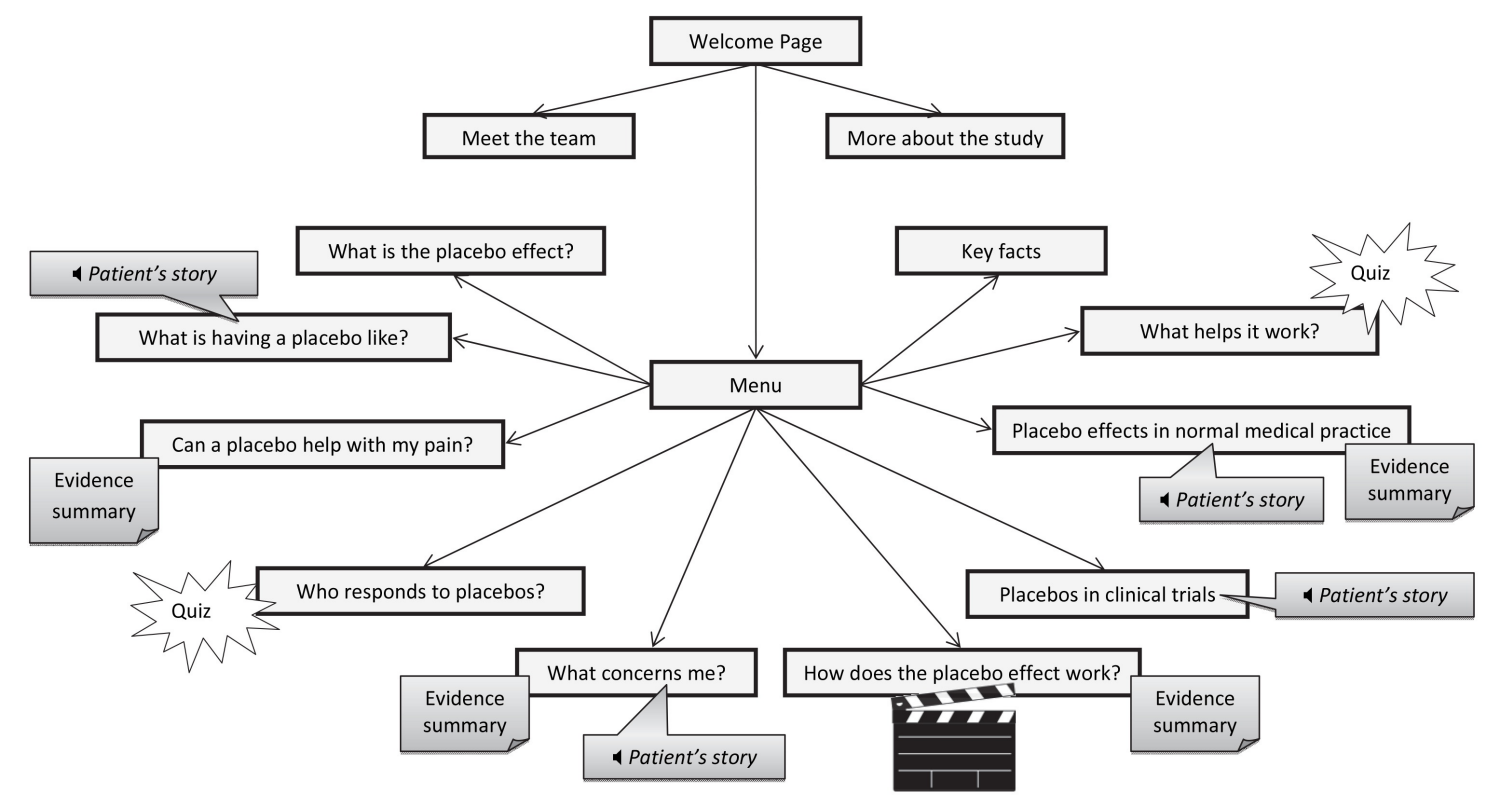
Overview of structure and contents of website.

**Figure 3:**
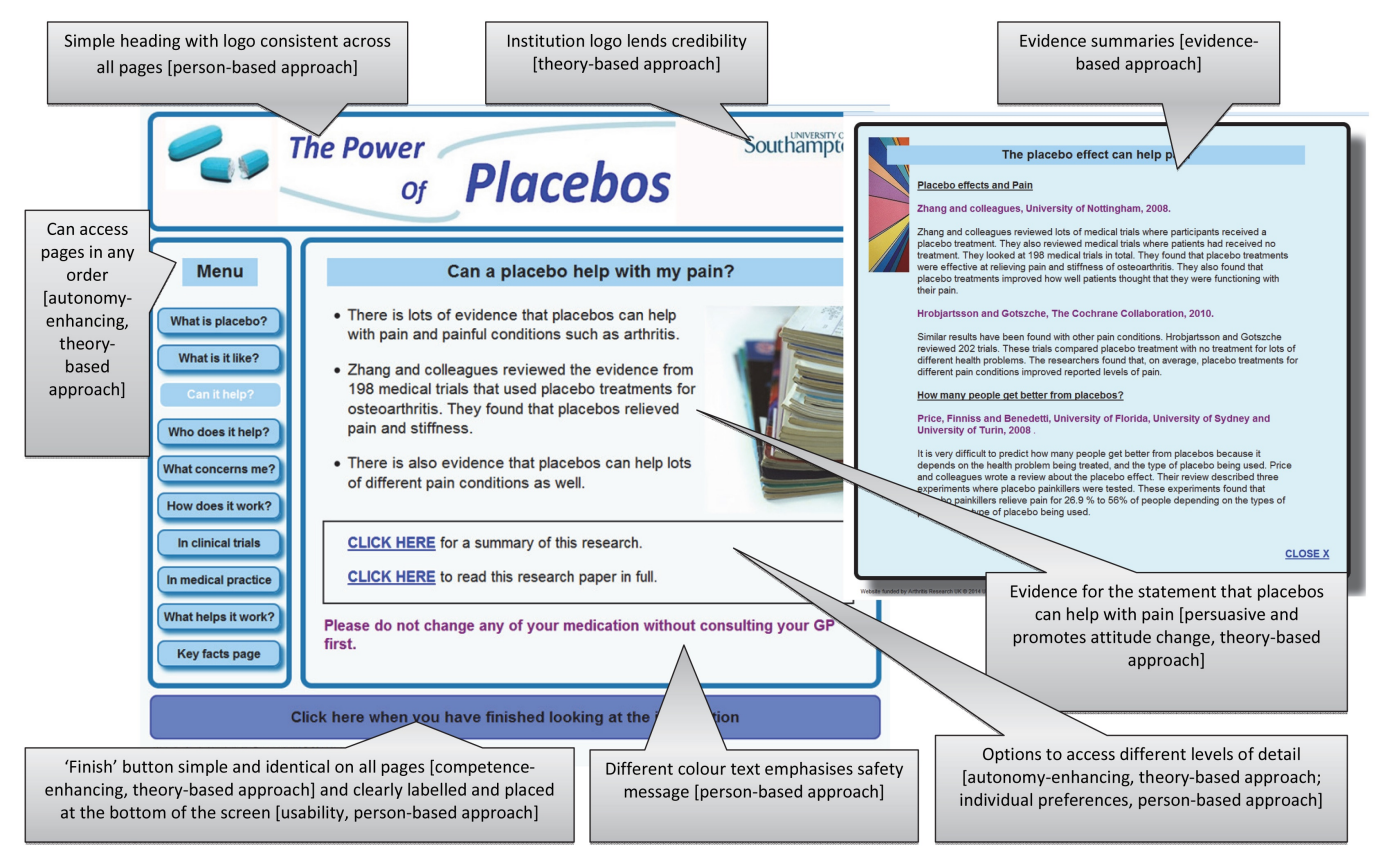
Example page annotated to highlight key features and their development from evidence-, theory-, and person-based approaches.

The final structure and main content of the website is shown in Figure 2. Nine topics are covered across 10 main pages (the last page offering a summary of key facts), which can be accessed in any order. Text and images are supplemented by scientific evidence summaries (on 4 pages), audio clips with photos and transcripts of patients’ experiences (on 4 pages), a film, and 2 quizzes (with immediate feedback). Figure 3 shows one page (“Can a placebo help with my pain”), annotated with key features that illustrate the contribution of evidence-, theory-, and person-based approaches.

## Discussion

### Summary

We have developed a new website about placebos that conveys scientifically accurate information in a way that should engage members of the public and enable them to make informed choices about placebos. The development process drew on evidence about placebo effects, theory about education, attitudes and motivation, and qualitative research to maximize the website’s likely effects.

### Strengths and Limitations

Strengths of this project include the multidisciplinary team and the combination of evidence-, theory-, and person-based approaches [28-30]. Team members shared expertise in topics, including placebo effects, digital interventions, film, and chronic pain, and contributed diverse professional perspectives (eg, psychology, general practice, physiotherapy, acupuncture). Two team members were patient representatives whose input helped ensure our website addressed important issues in an accessible and nonpatronizing manner. By drawing on a combination of approaches we were able to incorporate evidence, theory, and users’ perspectives in a flexible manner throughout the intervention development process.

The main limitation of the study was the homogeneous sample of participants. Involving a more diverse sample of patients in our think aloud study would have allowed us to better gauge the comprehensibility of the website among members of our target audience, adults with experience of pain symptoms. The same issues were raised repeatedly across the 10 interviews, suggesting additional interviews would have produced diminishing returns for additional cost. However, additional interviews with people with other characteristics (eg, more severe chronic pain conditions) might have elicited novel comments. While participants’ views on the format and structure of the website were vital for improving its usability, these issues may have been particularly pertinent for our sample because, from their comments, they appeared to be experienced Internet users with a good baseline understanding of placebo effects. A more diverse sample might have uncovered additional issues and/or misunderstandings about placebos and placebo effects. Future think aloud studies for website development would benefit from sampling diverse participants from the population of likely end-users and finding ways to encourage them to focus on the substance of the website as well as its style and structure. The full person-based approach includes guidance on how to achieve this to ensure qualitative research goes beyond user-testing to focus on participants’ perspectives on substantive issues [30].

### Applications

Using our website to inform potential clinical trial participants about placebos could have both beneficial and detrimental consequences. For example, it might improve the validity of informed consent [3], alleviate anxiety about placebos, ease the process of unblinding to placebo allocation at the end of trials [15,16], reduce adverse effects [62], and/or enhance recruitment [14]. However, increasing patients’ expectations of benefit during trials by encouraging positive beliefs about placebos might increase the size of the placebo effect [63,64]. This could introduce bias [65] particularly if placebo and intervention effects are not additive [66] and/or could reduce estimated treatment effects [11]. It is therefore vital to test the effects of providing comprehensive information about placebos before changing research practice. To contribute to this much-needed evidence base, we will report separately a randomized experiment testing the effects of our new website on knowledge and informed choice about placebos.

### Conclusions

In conclusion, we have developed an evidence-based website that incorporates theory-based techniques to inform members of the public about placebos and placebo effects. Before using the website in clinical trials it is necessary to test its effects on key outcomes, including patients’ knowledge and capacity for making informed choices about placebos.
